# The Performance of Inertial Measurement Unit Sensors on Various Hardware Platforms for Binaural Head-Tracking Applications

**DOI:** 10.3390/s23020872

**Published:** 2023-01-12

**Authors:** Petar Franček, Kristian Jambrošić, Marko Horvat, Vedran Planinec

**Affiliations:** University of Zagreb, Faculty of Electrical Engineering and Computing, Unska 3, 10000 Zagreb, Croatia

**Keywords:** binaural synthesis, head tracking, IMU sensors, virtual reality

## Abstract

Binaural synthesis with head tracking is often used in spatial audio systems. The devices used for head tracking must provide data on the orientation of the listener’s head. These data need to be highly accurate, and they need to be provided as fast and as frequently as possible. Therefore, head-tracking devices need to be equipped with high-quality inertial measurement unit (IMU) sensors. Since IMUs readily include triaxial accelerometers, gyroscopes, and magnetometers, it is crucial that all of these sensors perform well, as the head orientation is calculated from all sensor outputs. This paper discusses the challenges encountered in the process of the performance assessment of IMUs through appropriate measurements. Three distinct hardware platforms were investigated: five IMU sensors either connected to Arduino-based embedded systems or being an integral part of one, five smartphones across a broad range of overall quality with integrated IMUs, and a commercial virtual reality unit that utilizes a headset with integrated IMUs. An innovative measurement method is presented and proposed for comparing the performance of sensors on all three platforms. The results of the measurements performed using the proposed method show that all three investigated platforms are adequate for the acquisition of the data required for calculating the orientation of a device as the input to the binaural synthesis process. Some limitations that have been observed during the measurements, regarding data acquisition and transfer, are discussed.

## 1. Introduction

Auralization techniques based on binaural synthesis are penetrating many fields: the immersive virtual world of computer games and smartphone applications, the walk-through modes of architectural design programs, the design of acoustically sensitive spaces, and many more to come [1,2,3]. The combination of new acoustic modeling methods and immersive audio rendering techniques provides new auralization-based tools for perceptual assessment of acoustic impact in diverse environments [4].

Binaural hearing is essential for realistic auditory perception because it presumes the ability of the human hearing to perceive spatially separated sound sources. The Head-Related Transfer Functions (HRTFs) are essential input information for emulating spatial hearing. Many datasets of HRTFs are already available, and several methods have been developed for measuring personal HRTFs in an easy way [5,6]. Various studies show the advantage of using one’s own measured HRTFs in binaural systems [7,8], rather than relying on generic ones.

The most natural and immersive sound experience is achieved by coupling binaural systems with head-tracking devices. Another common tracker for body-part movements are the eye-tracking devices used in many computer-vision applications [9], or those that sense the position of a person’s finger for interacting with objects in virtual- and mixed-reality systems [10]. Many different systems are used for head-tracking applications: ultrasonic trackers, electromagnetic devices, optical analogue and digital systems, face-tracking camera systems [11], and, most frequently, inertial systems with up to six degrees of freedom (with and without optical reference) based on Inertial Measurement Unit (IMU) sensors [12]. The most advanced technology used for manufacturing the IMUs (triaxial accelerometers, magnetometers, and gyroscopes) is the space- and money-saving MEMS technology (Micro Electro-Mechanical System). The MEMS technology is particularly suitable for small integrated electronic devices, such as smartphones.

Not all of the listed types of sensors and systems are equally suitable for auralization purposes since the latency of the head orientation measurement system should be as low as possible. The transfer and processing of the read data must be fast enough in order not to create perceivable lags that disturb the perception of transparency and smoothness of the binaural image changes for the listeners in motion [13].

The quality and accuracy of the orientation estimation can be improved by sensor fusion and advanced filtering, such as the Multi-Rate Unscented Filter [14]. Head-movement trackers are used not only in binaural synthesis, but also in many other, often health-related studies, services, and applications [15,16]. Some authors have used deep-learning algorithms to improve the real-time detection of the head orientation or strive to detect the pose of the whole body by using multiple IMU sensors [17,18]. Dedicated commercial binaural systems with head tracking are still quite expensive, as they are not produced in large quantities and require several sensors and a computer unit used solely for performing this task [19].

**Figure 1:**
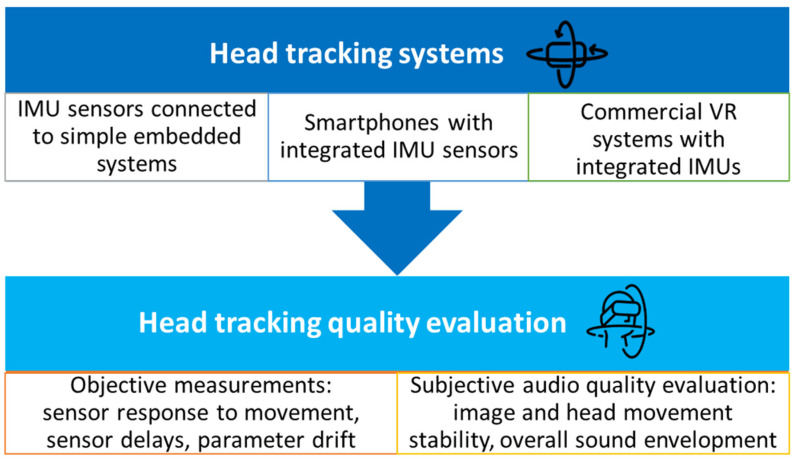
The outline of the AUTAURA project: quality evaluation of head-tracking systems based on IMU sensors.

Ideally, when these devices are used for head tracking in auralization applications, they should move in perfect synchronicity with the user’s head. To achieve this, the tracking device must be fastened to the head in a simple and efficient manner—usually either as a part of the headphones or as a part of the headset used in a Virtual Reality (VR) system. The goal of this research is to determine and compare the objective quality of the IMU sensors in certain types of devices that can be used for head tracking to aid binaural synthesis: embedded systems (such as Arduino-based boards, with integrated or added IMU devices), smartphones (Android of iOS based, with already integrated IMUs), and a commercial VR system (also with integrated IMUs) [20]. The output data obtained from these head-tracking devices are their calculated orientation in space with three degrees of freedom (DoF): Roll, Pitch and Yaw [21,22,23]. The additional three degrees of freedom (adding up to a 6-DoF space) track the translation of the device along the x-, y-, and *z*-axis, thus needing a GPS tracking sensor. Figure 1 shows the outline of the AUTAURA research project, and this paper presents a part of the research that has been carried out within the scope of this project [24].

## 2. Measurement Challenges

The measurements performed on IMU sensors can be divided into two main groups: (a) static measurements of parameter drift that track the changes in the output of the IMU sensors over time when the sensors are not in motion; and (b) dynamic measurements of parameter precision, time lag, and drift over time while the sensors are moving or being reoriented at some reference position.

This paper describes the dynamic measurements on IMU sensors integrated in different hardware platforms. To account for the ways these platforms transfer the IMU sensor data to the user, the measurement process must be set up and adjusted individually for different platforms. In doing so, many challenges and limitations arise that are related either to the platform itself, or to the quality of the built-in IMU sensors. A detailed description is given in the subsections that follow.

### 2.1. Data Output on the Investigated Hardware Platforms

All the investigated hardware platforms offer wireless data transfer using the Wi-Fi protocol or the BLE (Bluetooth Low Energy) protocol, or both. The issue with both protocols is that the data-transfer speed they provide is not high enough, thus introducing a time lag in the sent data. This time lag is hard to control, and it is difficult to know its exact value, meaning that the data measured on different platforms cannot be compared.

The Arduino boards were equipped either with built-in IMU sensors, or with external sensors connected to their pins. Dedicated codes have been written to collect the data at the highest possible sampling rate and send them to a computer, using serial data transfer via USB connection at 115,200 bauds. The data were saved on the computer in files with a relative timestamp that shows the elapsed time in milliseconds, which are the smallest possible time-increment measurement for data collection in the Arduino system.

The data provided by the built-in IMU sensors in smartphones were collected by using the MATLAB Mobile application [25], designed and produced by Mathworks, Inc., located in Natick, MA, USA. It offers double functionality: (a) raw data acquisition from IMU sensors (triaxial acceleration, angular velocity, and magnetic field) and (b) simultaneous calculation of the orientation of the IMU sensor (and, consequently, of the phone itself), using a preprogrammed function. The resulting orientation is given in angular coordinates of yaw, pitch, and roll. The IMU data can be sampled at arbitrary sampling frequencies up to 100 Hz.

Finally, a program was coded in the Unreal engine developer app [26] with the function of collecting the data from the IMU sensor of a VR headset and sending it to a computer via USB connection to be logged in a file. Angular acceleration, linear acceleration, angular velocity, and linear velocity were recorded together with their corresponding event tick timestamp. The sampling frequency was determined by the operating system of the device and was set to 60 Hz. Additional complex coding would be needed to change the sampling rate, and this was deemed to be out of the scope of this paper.

To ensure a fair comparison of the performance displayed by all the investigated hardware platforms, the raw data collected from all hardware platforms were postprocessed by using the same MATLAB script for calculating the orientation of the devices.

### 2.2. Sampling Frequency Variability and the Update Rate of IMU Sensor Data

The first problem that was found was the variability of the set sampling frequency. To compare data across platforms, it was important to sample sensor data at the same time instants. The Arduino-based devices utilized various IMU sensors made by different manufacturers. They usually provide data at the rate (the sampling frequency) set by their IMU components. The magnetometer usually has a lower maximum sampling frequency than the other two sensors do. For example, the maximum sampling frequency for the accelerometer and gyroscope for Arduino Nano 33 BLE is 104 Hz, but it is only 20 Hz for the magnetometer. If it is to be used in calculations of the orientation using the MATLAB script, the sensor data sampled with a lower sampling frequency than the rest of the dataset must be upsampled. However, the upsampling process does not provide new sensor data. Instead, it just predicts the values at time instances when there was no actual data coming from the sensor, thus potentially decreasing the accuracy of the raw data measurements and, consequently, of the calculated orientation.

**Figure 2:**
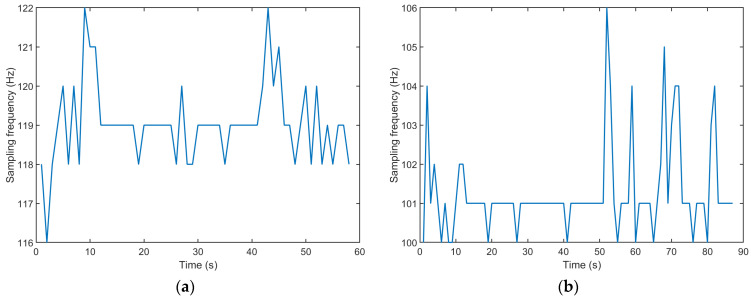
An example of sampling frequency variability in measurements on (**a**) Arduino-based devices with IMU sensors and (**b**) smartphones with IMU sensors.

Moreover, the data sampling rate for Arduino-based devices is set by the memory buffer of the sensors that stores the current values. If the values are read faster than they are updated, the same numerical value will be read repeatedly until it has changed. Therefore, the true sampling frequency of the IMU chip can only be guessed. A simple count of the number of updated sensor values in each 1-second period (when there is a change in its numerical value) shows that this number is not constant, as shown in Figure 2a. This automatically increases the uncertainty of the calculated orientation.

In the measurements performed on smartphones, the MATLAB application logs the data with a unique timestamp for each measurement parameter. The analysis of the data collected from some of the devices reveals a discrepancy between the chosen sampling frequency and the actual time interval between individual samples. Specifically, the samples are taken with a time interval of 7 ms, implying that the real sampling frequency is around 142 Hz. Moreover, the sampling frequency on smartphones (when numerical changes in sensor values can be observed) also deviates from the set constant value, as shown in Figure 2b. Again, this increases the uncertainty of the measured orientation of the smartphones.

To avoid some of the problems caused by different sampling frequencies, all data can be upsampled to a unique sampling frequency (e.g., 200 Hz), and linear interpolation can be used to calculate the values of the new samples. This solution allows the measured sensor data obtained from all devices to be directly compared due to the common upsampled time reference.

### 2.3. Time Base Mismatch

Another important source of measurement uncertainty is the mismatch in the time base of the collected data that is recorded in the form of timestamps. The timestamps are used to determine the actual sample rate and to synchronize the data obtained from different devices. Each measured device has its own clock generator that is also used for writing the timestamp. If there is a clock mismatch between devices, the orientation results obtained for a device will be compressed or expanded in time compared to other devices, thus introducing a measurement error.

An issue with the time-base mismatch occurred during the measurements on one of the smartphones. The inspection of the data revealed that the MATLAB Mobile application wrote the timestamps that ultimately yielded a total measurement duration that was shorter than the actual one. This type of error is very easy to detect if the angular movement trajectory calculated for one device deviates from the ones calculated for other devices. On the other hand, if several devices exhibit a time-base mismatch, its detection becomes rather difficult, as it is impossible to declare any device as the one that provides the absolute time reference.

### 2.4. Missing or Imprecise Data

**Figure 3:**
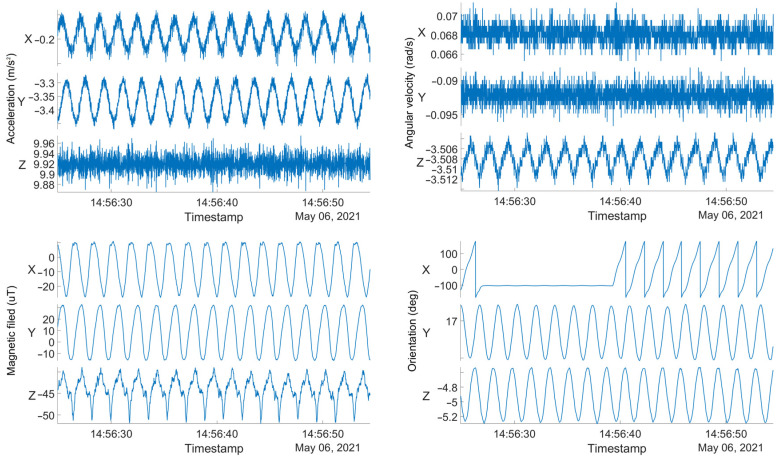
An example of the measured raw dataset from the IMU sensors on a smartphone. From left to right and from top to bottom: triaxial acceleration in m/s^2^, angular velocity in rad/s, magnetic field in µT, and orientation (yaw, pitch, and roll) in degrees.

In certain cases, the measured data obtained from sensors cannot be used to calculate the orientation of a device. Figure 3 shows an example of raw data obtained from all three triaxial sensors in a smartphone (accelerometer, gyroscope, and magnetometer) placed on a turntable rotating at 33 RPM. The orientation calculated from these data by the internal algorithm is shown as well. It can be seen that the calculated orientation in the *x*-axis does not consistently follow the actual rotational movement of the device at certain time instances, thus introducing another source of error.

**Figure 4:**
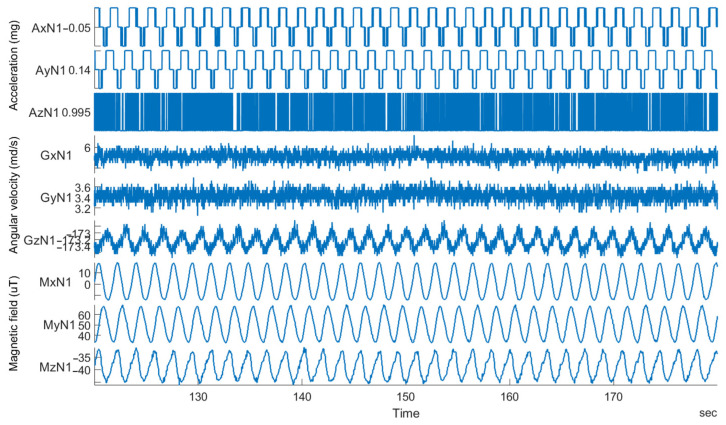
An example of measured triaxial raw IMU data on an Arduino-based device—from top to bottom: acceleration in mg, angular velocity in md/s, and magnetic field in µT.

Similarly, an example of the raw data measured on an Arduino-based IMU device with the same triaxial sensors is shown in Figure 4. A strong granulation of some of the measured data can be seen in comparison to the data obtained for smartphones, particularly for the accelerometer, which again leads to a greater error in the calculated orientation of the device. The reason for this is the IMU sensor itself being forced to read the data with amplitudes that are clearly below its optimal operating range. In many cases, especially for cheap sensors, the operating range is not even listed.

### 2.5. Magnetic Field Disturbances

For all intents and purposes, in the context of this research, the magnetic field of the Earth is constant and always oriented from north to south. Therefore, it can be used as a reference for the IMU sensors in order to prevent drift and/or provide the input for periodical self-calibration of the entire IMU system in a device. However, this magnetic field is rather weak and can be easily disturbed or completely overpowered by other sources of magnetic fields, typically electrical motors or wireless transmitting devices [27,28,29]. Unfortunately, this kind of influence can hardly be anticipated, as humans do not have a sense that reacts to magnetic fields.

**Figure 5:**
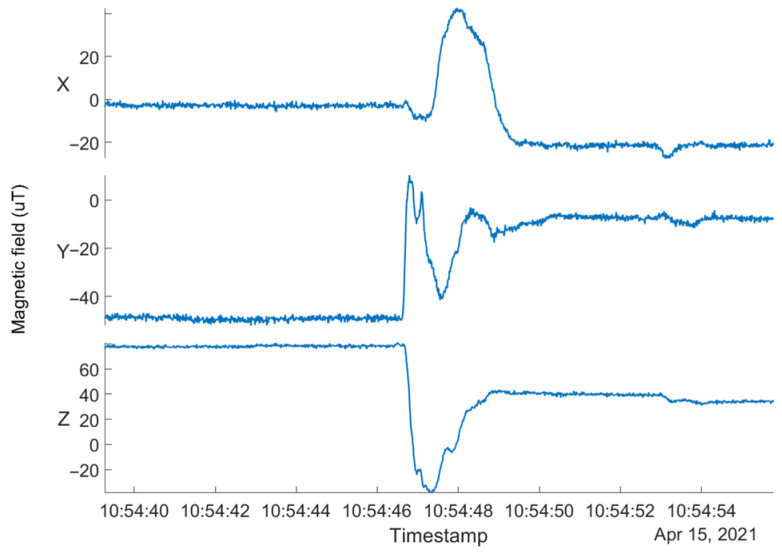
Triaxial magnetometer data before and after switching on the electric motor of the rotating platform. The initial disturbance in the magnetic field is caused by switching on the electric motor. With the platform in operation, the magnetometer data exhibit a permanent shift in all three axes.

This phenomenon was observed during the dynamic measurements performed as part of the previous research [30]. Figure 5 shows an excerpt of the measured magnetometer data for a smartphone mounted on a platform rotating at 0.75 RPM. The sharp spike in the magnetic field in all three axes occurred at the instant when the motor was switched on and the platform started to rotate. Moreover, a constant shift in the magnetic field remains in all three axes once the initial disturbance caused by switching the motor on has stabilized. In this case, the error in the magnetic field readings is too great, and the magnetometer data can no longer serve as reference and/or the source for calibration.

More information can be collected if the position of the smartphone is calculated by using GPS sensors. However, this approach was not implemented in this research since (a) not all devices were equipped with GPS sensors, and (b) GPS sensors operate very poorly in closed spaces (or not at all).

These findings suggest that the magnetometer readings do not necessarily improve the accuracy of the calculated orientation of a device if external magnetic disturbance is present. As it is difficult, if not impossible, to predict, detect, and compensate for such disturbance, it is probably wiser to disregard the magnetometer data altogether. The downside of this approach is the loss of any possibility of periodic self-calibration of the orientation of the device.

**Figure 6:**
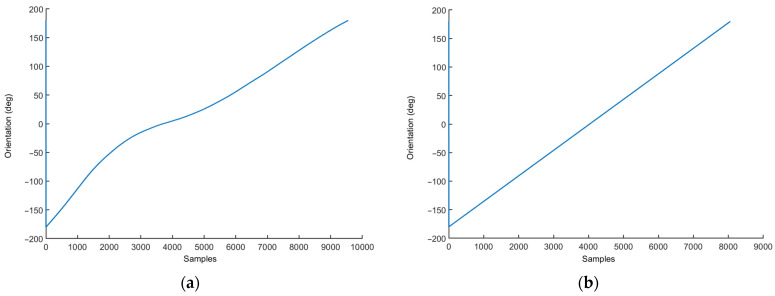
The orientation (azimuth) of a smartphone placed on slowly rotating, electric-powered platform, calculated by a MATLAB script, using (**a**) all sensor data and (**b**) only the accelerometer and gyroscope data, but not the magnetometer data.

A simple confirmation of the proposed course of action can be seen in Figure 6, which displays the calculated orientation of a smartphone placed on a slowly rotating, electric-powered platform, as described above. It is visible that the orientation of the device is more accurate when magnetometer data (shown in Figure 5) are omitted from the calculations due to great magnetic disturbance caused by the electric motor.

## 3. Previous Research

The authors of this paper conducted several pilot measurements on IMU sensors that helped in designing the new measurement method presented in Section 4. Table 1 gives an overview of the previous measurements, which are described in more detail in the following subsections.

### 3.1. Static Measurements

It is difficult to assess the overall quality of IMU sensors based only on dynamic measurements with the duration of mere seconds. Such measurements are not long enough to reveal the drift of the measured parameters that can develop over time. To expose potential drift problems, especially on low-quality sensors [32,33], long-term static measurements (duration in minutes or even hours) are performed on IMU sensors on various hardware platforms, with no movement allowed.

The Allan variance is a proven tool for determining sensor reliability, especially when the parameter drift over time is considered [34]. Certain types of variance curve slopes can reveal the behavior of the device, with the typical ones being −1, quantization noise; −1/2, angle (velocity) random walk; +1 and 0, bias instability; +1/2, rate random walk; and +1, drift rate ramp. Moreover, the results of analyses by means of Allan variance start to behave very similarly once the sampling rate of the sensors has been increased above 50 Hz [35].

**Figure 7:**
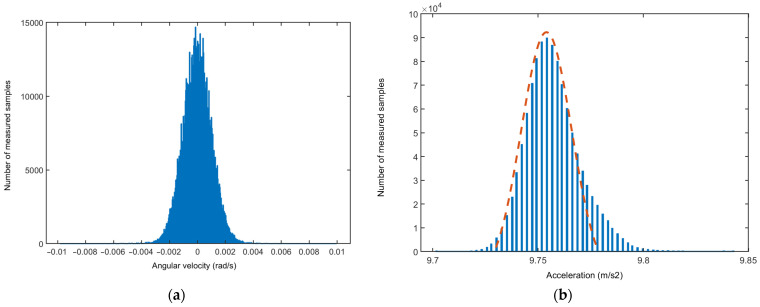
(**a**) The histogram of the *x*-axis angular velocity measured with the IMU sensor in a smartphone. (**b**) The histogram of the *z*-axis acceleration measured with the IMU sensor in the same smartphone. The orange dashed curve with normal Gaussian distribution is added for comparison, revealing the skewness of the acceleration data.

In the previous research [31], the static behavior of IMU sensors built into Arduino-based systems and smartphones was investigated on a selected group of devices by measuring the Allan variance for all available triaxial IMU sensors. The resulting histogram of the measured data received from a sensor can already reveal potential problems with a sensor unit. The expected distribution of data should be a Gaussian-type one, as illustrated in Figure 7a. Any skewness in the distribution points to an irregular behavior of the sensor during the measurement period. An example is shown in Figure 7b.

**Figure 8:**
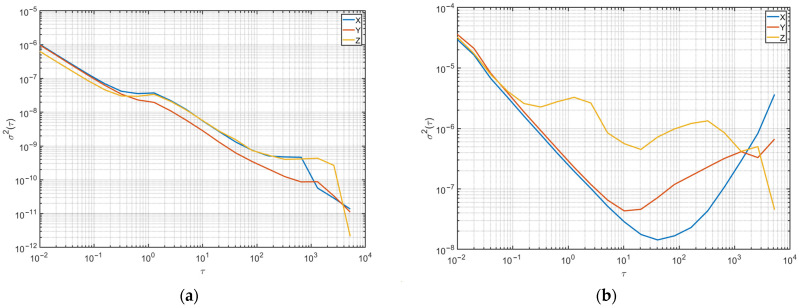
(**a**) Allan variance vs. time for angular velocity data obtained from a smartphone—no indication of a drift as the value of the variance is constantly decreasing. (**b**) Allan variance vs. time for acceleration data obtained from the same smartphone—a drift starts to occur after 40 s for the *x*-axis data, and after 10–20 s, it occurs for the *y*-axis data, as the variance starts to increase again after reaching its minimum value.

A more definitive diagnosis can be made by plotting the Allan variance against the logarithm of the elapsed measurement time, as displayed in Figure 8 for one of the investigated smartphones. The plot in Figure 8a shows no drift of the angular velocity during the measurement period. On the other hand, the plot in Figure 8b shows a local minimum of the Allan variance for acceleration in both the *x*-axis and *y*-axis that occurs after a certain amount of time has elapsed. The increase of the Allan variance with time as the measurement goes further is a clear indication of a drift.

Although static measurements and analyses can reveal many issues that affect the behavior of an IMU sensor, the accuracy and precision of the orientation data are even more important in applications where the device is in motion and its true orientation is critical. One such application is binaural synthesis with head tracking.

### 3.2. Dynamic Measurements

**Figure 9:**
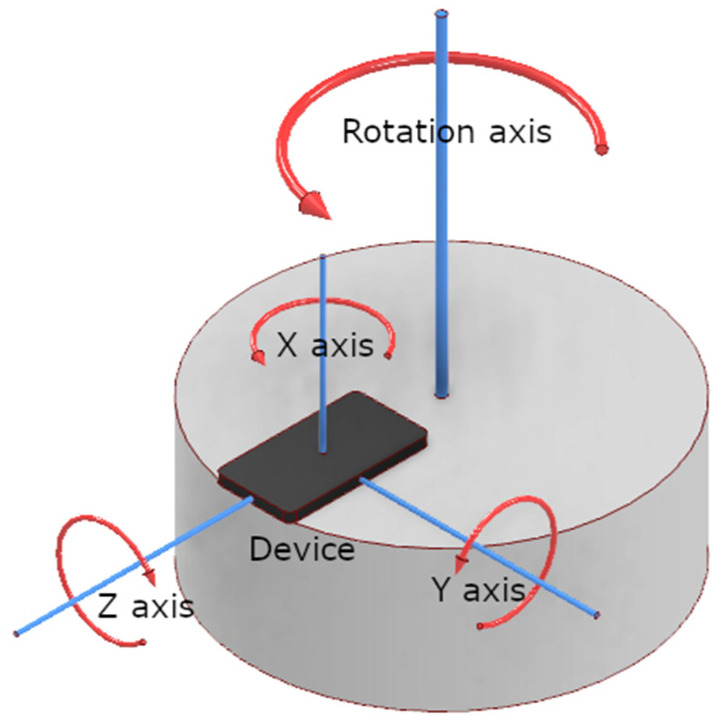
The principle of dynamic IMU measurements implemented in previous research (measurements on platforms rotating at constant rotation speed).

In the previous research [30], dynamic measurements were conducted on IMU sensors built into various Arduino boards and smartphones. The principal setup for these measurements can be seen in Figure 9.

Two motorized rotary platforms were used to provide uniform and equal movement for all of the investigated devices. The main requirement for this research was to provide a constant and stable rotation speed of such platforms so that the real orientation of all measured devices could be known at any given time. To conduct the measurements at different rotation speeds, one platform was rotating at a rotation speed of only 0.75 RPM, whereas the other one was essentially a turntable with a considerably higher rotation speed of 33 RPM. The two platforms were used to investigate the accuracy of orientation calculations for the measured devices in both slow- and fast-moving conditions.

**Figure 10:**
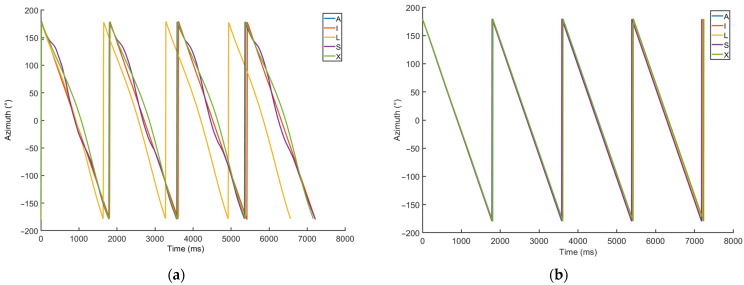
The comparison of the calculated azimuth for all smartphones investigated, using the platform rotating at 33 RPM: (**a**) internally precalculated by using the raw data from all sensors; (**b**) calculated by using a MATLAB function, using only the accelerometer and gyroscope data.

The selected results of these measurements can be seen in Figure 10. Figure 10a shows the orientation (azimuth) calculated for five smartphones using the built-in algorithms. A clear difference can be seen between one device and the rest of the devices, due to a significant time drift exhibited by that device. Additionally, the azimuth calculated for some devices does not change in a strictly linear fashion, as would be expected, given the constant and stable rotation of the rotating platforms. Figure 10b shows an improvement in the accuracy of the azimuth calculated from raw data for the same devices, using a dedicated MATLAB function. The improvement was achieved by not using the magnetometer data that were corrupted by the magnetic field generated by the electric motor of the rotary platform.

The main limitation of these dynamic measurements was that the rotation speed of the platforms is constant. As such, this measurement setup is not fully representative for real-life applications in binaural synthesis with head tracking. In such applications, one can expect that the IMU devices will constantly change their orientation as the user’s head moves. The head movement is erratic and unpredictable and is usually limited to a confined spatial angle (unless the user’s entire body is in motion). Another drawback of the rotating platform setup was the inability to use wired connections between the measured devices and the data-acquisition computer. To improve the measurement procedure and overcome these drawbacks, a new measurement method is proposed, as described in detail in the following section.

## 4. The Proposed Measurement Method

### 4.1. Requirements

The design of the new measurement method needs to facilitate dynamic measurements with a variable angular velocity that can be applied to different sensors and devices. Certain similarities can be found with the research described in [36]. In general, the utilization of a highly accurate and precise robotic system with three or even six degrees of freedom (tracking both the orientation and the position of the IMU unit) leads to highly accurate measurements [37]. However, the cost of such a setup makes it practically unavailable to a broader community.

**Figure 11:**
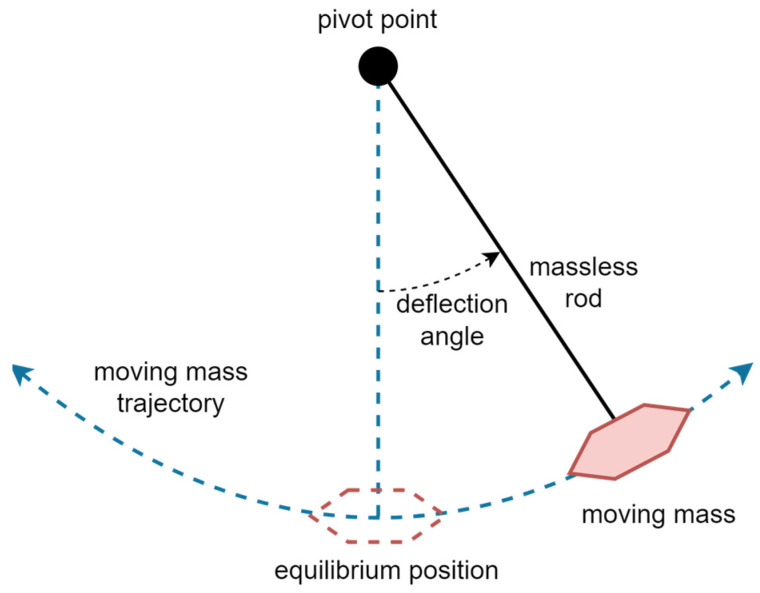
The ideal gravity pendulum [40].

To assure the highest possible accuracy and to minimize measurement errors, specific demands must be addressed:

- Extra consideration is given to the comparability of the measured results. Therefore, the measurements must be conducted simultaneously for many different sensors and/or devices. Furthermore, the sensors and devices should be positioned in a manner that will ensure that their output data meet the given criteria for a direct comparison of the results.
- The measurement setup needs to accommodate different shapes and types of sensors and devices.
- Different hardware platforms and devices under investigation lead to the problem of the time synchronization of collected data. While some of the measured devices have the possibility to provide information on global time, thus avoiding the need for synchronization, other devices do not have this functionality. In general, the internal clocks of the investigated devices are not synchronized, thus making it necessary to employ a synchronization mechanism of some kind.
- For real-time data acquisition, communication should be robust and stable. Wireless communication does not meet this requirement and introduces a lag in the data transmission. Wired communication is preferred for this reason, especially because it is also used to power the devices.
- Besides acceleration and angular velocity, the data obtained from magnetometry are often used to calculate the orientation of a device. To ensure that the possibility of generating disturbing magnetic fields is as low as possible, the use of electromechanical (motors) and wireless devices should be avoided altogether.
- As the budget for the measurement setup based on this method is intentionally restricted, the use of advanced and expensive equipment is not allowed.
- A possible solution that meets these specific demands is a setup that utilizes a simple mathematical pendulum (gravity pendulum). Similar solutions have been implemented in [38,39]. An example of a gravity pendulum is shown in Figure 11.

Although the sensors cannot be placed in such a way as to occupy the same position in space, they can be positioned in such a way as to avoid the influence of the difference in positioning on measured data. It is possible to construct a setup in which multiple and generally different devices can be positioned with different orientations, as well as in different orders and locations, so that the collected data are simultaneously recorded for all of them during the same measurement. Time synchronization is achieved by generating a strong mechanical pulse recorded on all sensors before and after each measurement. As the desired movement is facilitated by the Earth’s gravity, no motors or any type of actuators are needed. Thus, they are eliminated as potential sources of magnetic disturbance. The pendulum is placed in an ambience without magnetic sources to minimize magnetic interference. The small range of motion of the pendulum and its design allow the use of wired connections to the sensors. The cost of this laboratory setup remains within the allotted budget.

### 4.2. Measurement Setup

**Figure 12:**
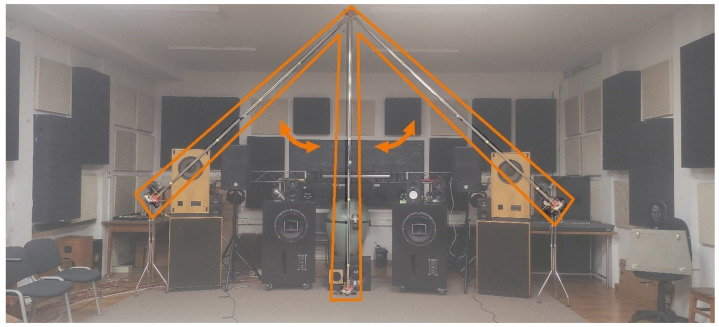
Measurement setup based on the gravity pendulum. Maximum displacement positions on both sides and the equilibrium position in the middle are marked.

The laboratory setup was assembled by using 3D-printed ceiling brackets as the pivot point, a long aluminum rod, a wooden platform at the end of the rod that was used for placing the sensors onto it, and an additional weight placed just below the platform. A computer with corresponding wiring was used for communicating with the sensors and for powering them. The plastic brackets were mounted on the ceiling of the laboratory at the height of 3.2 m. A small metal axle spanning between the brackets forced the pendulum to swing in a single plane. The rotational friction that appears during the movement of the pendulum (and its axle) was minimized by using a pair of high-class ball bearings at the ends of the axle. The rod was created from a 3 m long piece of U-shaped aluminum profile with a thin cross-section. This shape was used to ensure the simple and easy installation of the wiring and to maintain the stiffness of the rod. The wooden platform was fixed to the rod by means of a plastic 3D-printed fastener. The platform needs to be perpendicular to the rod, so that the radius of rotation is the same for all measured sensors. As the mass of the wooden platform is rather small, a steel weight was added below it to increase the moving mass and to lower the center of mass of the entire pendulum. A higher mass leads to higher kinetic energy of the pendulum, thus yielding smoother recorded data, even at a higher inclination from equilibrium. Moreover, the added mass facilitates a longer measurement duration, as it enables the pendulum to swing for a greater number of periods before coming to a stop. The described laboratory setup is shown in Figure 12.

Figure 12 shows the setup with three critical positions of the pendulum. In the two positions with maximum inclination (left and right position), the acceleration of the moving mass has its maximum, and its angular velocity is zero. In the equilibrium position, the moving mass is at its lowest point, the angular velocity is at its maximum, and the acceleration is zero.

It is important to note that all the loudspeakers visible in the background were moved far enough from the laboratory setup to minimize the influence of any generated magnetic field.

### 4.3. Selection of Three Hardware Platforms

The measurements made within the scope of this research, using the described measurement setup, focused on three independent and, nowadays, often-used hardware platforms:

- Five do-it-yourself (DIY) IMU sensors. Three are connected to Arduino-based embedded systems, and two are an integral part of Arduino boards. Microcontrollers and sensors are labeled as follows: LSM6DS3 (external sensor module), NanoInt (internal), UnoInt (internal), ICMAK (external), and DOFv2 (external). The labels correspond to their IMU sensor chip, respectively: LSM6DS3, LSM9DS1, LSM6DS3, ICM-20600, and MPU-9250.
- Five smartphones made by different manufacturer brands and in different price ranges. The models used were Samsung S21, LG G6, Apple iPhone 12S, Xiaomi POCOx3NFC, and Samsung A50, and they were labeled S21, G6, iPhone, POCO, and A50, respectively.
- One headset for virtual reality, with unrestricted access to raw IMU data, labeled as VR. The brand of the device is Oculus Quest2, designed and produced by Meta, located in Menlo Park, CA, USA.

### 4.4. Data Sampling

The frequency at which the data were sampled was chosen as the maximum sampling frequency at which the accuracy of the sampled data is maintained. The sampling frequency depends on the type of measured parameters and on the type of hardware that is being measured. The DIY sensors offer the sampling frequency for acceleration and gyroscope data in the range from 70 to 150 Hz, for smartphones it is around 100 Hz, and for VR it is 60 Hz. The VR system is closed by design, and changing its sampling frequency represents a comprehensive task that is out of the scope of this research.

### 4.5. Uniformization of Output Data and Coordinate Systems

The devices and sensors that were investigated in this research do not have a unique and uniform format for the output data. For instance, the angular velocity can be expressed in degrees per second, but also in radians per second. Acceleration can be expressed in m/s^2^, but also in *g*. Furthermore, the VR device normalizes the data for acceleration; that is, in static measurements, its output is zero for all three axes, even though gravity is present. All other devices provide acceleration data that reflect the gravitational acceleration of the Earth. In addition, the position and orientation of the IMU sensors within a device with respect to the orientation of its casing is not straightforward and obvious.

In the absence of technical information and documentation about the investigated devices regarding the IMUs, the reference coordinate system needs to be determined for each device before any measurement can commence, and the output data from all devices need to be uniformized to be suitable for direct comparisons and for subsequent calculations. In other words, sensor readings obtained from different devices need to be comparable. To do so, the investigated sensors and devices are positioned still in static conditions, with only the gravity of the Earth present. A set of measurements is made for each device, and the orientation of the device is changed from one measurement to the next, so that all three axes are adequately covered. Magnitude correction factors are calculated from the obtained data for all three axes of each sensor.

In the final stage of the processing of the raw data, the corresponding correction factors are applied to raw data obtained from each sensor, so that both the raw data and the calculated orientations can be directly comparable.

### 4.6. Data Preprocessing

**Figure 13:**
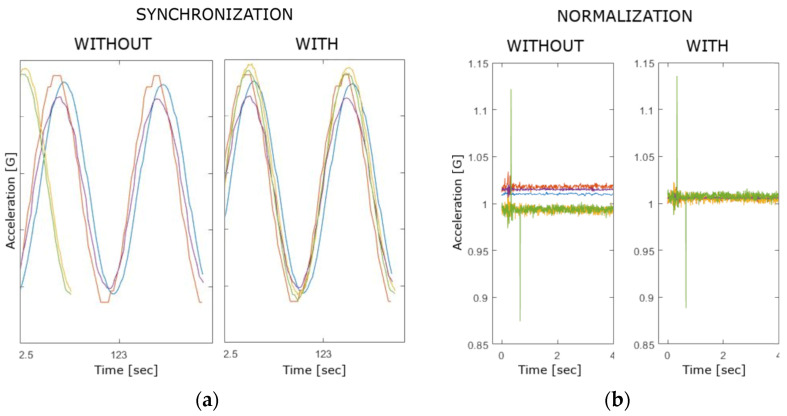
Comparison of raw accelerometer data measured on the *x*-axis without and with preprocessing: (**a**) synchronization and (**b**) normalization.

To compare the data obtained from different devices, a preprocessing procedure needs to be implemented prior to calculating the device orientations. As all the different devices being tested have different internal clocks, the timestamps of the measured data are generally incomparable. To overcome this issue and to synchronize all the data, all the devices were time-aligned by using a strong mechanical impulse both at the start and at the end of each measurement. The effect of such a synchronization can be observed by using the graphical representation of the measured data. Without it, the data coming from different devices would neither start nor end at the same moment in time, thus making the results incomparable. The timestamp differences observed without and with the implemented synchronization are shown on the chart in Figure 13a.

The normalization procedure is the second part of the preprocessing stage, and its goal is to align the amplitude of raw sensor data obtained from different devices. This procedure eliminates the bias (offset). The result of this procedure is displayed on the chart in Figure 13b.

The described preprocessing applied on measured data facilitates the direct comparison of different devices and makes all subsequent calculations considerably simpler. Nevertheless, all raw data collected during the measurements were saved in a unique database in their original form for further calculations and manipulation.

### 4.7. Calculation of the IMU Device Orientation

To facilitate a direct and absolute comparison of both the raw IMU data obtained from the investigated sensors/devices (acceleration, angular velocity) and the orientation data calculated from them, the devices would need to be placed on the test platform of the pendulum in a very particular way. Specifically, the IMU sensors in these devices would need to be aligned along the line that is perpendicular to the plane in which the pendulum swings. Such positioning would ensure that all the sensors exhibit the exact same range of motion. In addition, the physical orientation of the sensors in the devices would need to be in alignment in all three axes, meaning that the coordinate systems of all sensors are a perfect match to one another.

In reality, the position and the orientation of IMU sensors inside the devices they are built into (smartphones and VR systems) are generally unknown. Even when the orientation of the sensors is well-documented and known, it is extremely difficult to position and orientate the devices as described above. There is a very real possibility of a slight misalignment in positioning and/or orientation of the devices and the sensors inside them.

Rather than raw IMU data, the orientation of a sensor/device is the input that is needed for binaural synthesis and virtual reality applications. Therefore, instead of comparing the raw data themselves, the orientation of the investigated devices was calculated from them and subjected to comparison. The literature [22] provides an overview of algorithms that are used for calculating the orientation data. An orientation estimation based on quaternions is described in [41], and an orientation calculation based on an upgrade of the Kalman Filter is presented in [23]. In this research, the orientation of the investigated sensors and devices was calculated from raw IMU data (acceleration and angular velocity), using a dedicated MATLAB function [42].

The comparison of the calculated orientation facilitates the detection of possible differences in the placement and/or orientation of sensors during the measurements. As none of the devices can provide an absolute reference for either the position or the orientation, the data comparison would allow the operator to match the positions and/or orientation of the devices relative to one another in a series of repeated measurements. To avoid this lengthy and time-consuming process, the decision was made to take the starting orientation calculated for each device in the starting point of the pendulum as its individual reference. The orientation data calculated for each device over the course of the measurement were monitored and logged relative to the individual reference for that device. The relative orientation obtained in this way for all devices was then compared to determine if there are any actual differences between the devices.

## 5. Measurement Results and Discussion

This section presents and discusses the measurement results obtained from using the proposed measurement method. Comparisons are made within the groups of devices of the same type, as well as between different types of devices. The results obtained on the do-it-yourself (DIY) sensors are shown first, followed by the results obtained for smartphones. At the end, the comparison of one DIY, one smartphone, and the VR headset is presented. The measurement results are saved as raw data for each measurement, and the orientation is calculated in the postprocessing stage using a dedicated MATLAB function (as mentioned above) to facilitate a direct comparison of all types and combinations of devices.

The measured data include the internal time indicated by each device, the accelerometer data, and the gyroscope data. Each device was adjusted only to read and send raw sensor data without any additional computation performed, if possible.

### 5.1. Comparison of the Measured Gyroscope and Accelerometer Data and the Calculated Orientation

**Figure 14:**
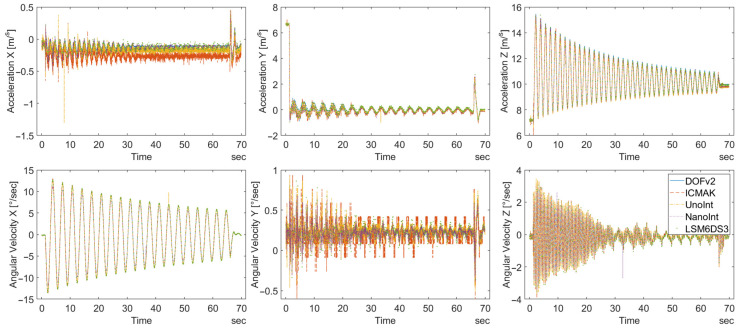
Raw accelerometer and gyroscope data in all three axes, measured using the DIY sensors.

This subsection shows the raw data obtained from measurements and the orientation calculated from these data. Figure 14 shows the raw data measured with DIY sensors for the full duration of the measurements.

**Figure 15:**
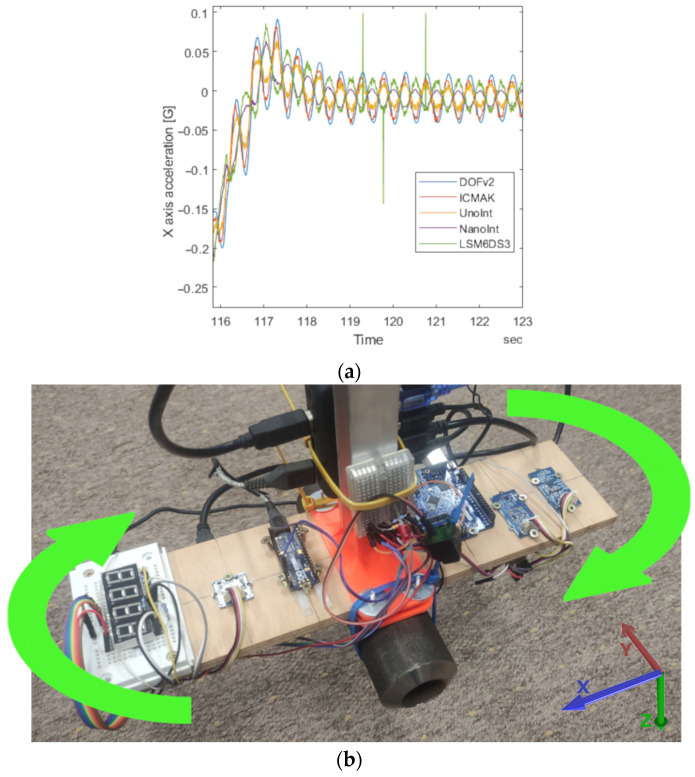
(**a**) Out-of-phase acceleration on the *x*-axis in the period of harmonic axial rotation. (**b**) The direction of the axes in laboratory setup and the torsional forces exerted on the wooden platform that carries the sensors.

The charts in the upper row of Figure 14 display raw data for acceleration. The directions of the axes correspond to the coordinate system displayed in the lower right part of Figure 15b. As expected, the largest amplitudes are found for the *z*-axis, while on the other two axes, the acceleration values are considerably lower, but not negligible. The gyroscope data were measured simultaneously with the acceleration data. As expected, the maximum amplitude of the angular velocity was found for the rotation along the *x*-axis. The bottom row of Figure 14 shows raw data obtained from gyroscope measurements for all three axes. Due to a slight torsional movement of the pendulum, the frequency of the torsional oscillation of the devices for the other two axes is much higher due to a significantly shorter radius of oscillation.

A closer view of the *x*-axis acceleration reveals that the data measured by NanoInt and LSM6DS3 are, in fact, out of phase with the data obtained from DOFv2, ICMAK, and UnoInt, as shown in Figure 15a.

The reason for the observed behavior is the torsional movement of the platform on which the sensors are mounted during the measurements, as indicated with green arrows on the photo shown in Figure 15b. The key fact for understanding the obtained data is that the NanoInt and LSM6DS3 sensors are mounted on the left side of the platform, whereas the remaining three sensors are mounted on the right side. The same discrepancy was observed in the acceleration data for the other two axes. On the other hand, the torsional movement of the wooden platform does not affect the gyroscope sensor readings. In this case, no out-of-phase readings were observed, and all rotations were in the same direction, as expected.

**Figure 16:**
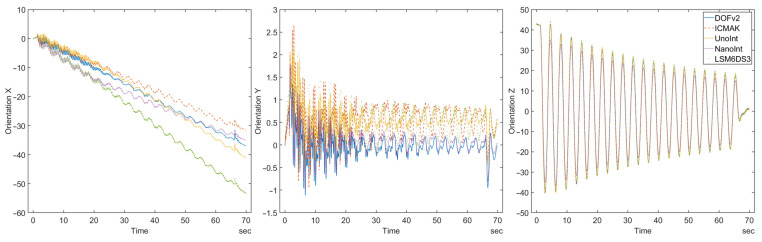
The orientation of the DIY sensors calculated in all three axes.

The comparison of the orientation calculated from the measured data reveals a behavior that can be explained as a digital drift. The orientation values calculated for the DIY sensors are shown in Figure 16.

As expected, the orientation on the *z*-axis displays the largest change, as shown in Figure 16. The described digital drift can be seen in the orientation data calculated for the *x*-axis. Due to torsional movement, low angular velocity is measured on the corresponding axis. As described above, measurements of small values are prone to a noticeable quantization error that affects all subsequent calculations. In Figure 14, it can be observed that the gyroscope data for the *y*-axis are not symmetrical around zero, although the torsional movement of the pendulum is symmetrical. The error cumulatively increases, leading to digital drift. To minimize the influence of digital drift in measurements with long duration, an automatic recalibration process needs to be implemented. Magnetometer data are usually used for this purpose. However, these data were not considered in this study, so the sensors could not be recalibrated. Fortunately, the accuracy of the calculated orientation was not affected by drift in any way, because the movement of the pendulum (and the sensors along with it) occurred in the yz-plane, whereas the observed drift was present on the *x*-axis. This method reveals an inherent drawback of IMUs: all measured sensors tend to drift when measuring a very low angular velocity.

**Figure 17:**
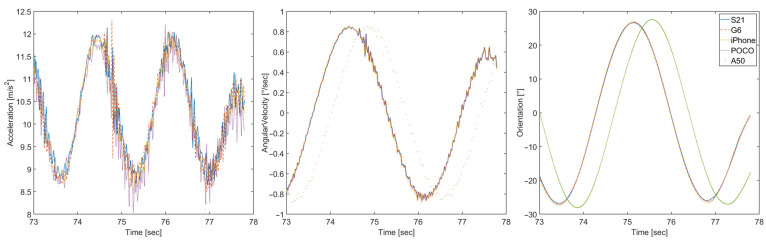
Acceleration on the *z*-axis (**left**), angular velocity on the *x*-axis (**center**), and orientation on the *z*-axis (**right**) obtained for five smartphones under test.

The measured data confirm the expectation that the acceleration on the *z*-axis and the angular velocity on the *x*-axis are the most relevant for describing the motion of the pendulum used in the test setup. Figure 17 displays the comparison of these two parameters and the calculated orientation for five investigated smartphones.

Figure 17 shows the presence of glitches and spikes introduced by the measured smartphone devices into the accelerometer data. The gyroscope data are considerably smoother, but the A50 smartphone displays a time latency of around 500 ms compared to other devices. This latency is reflected in the calculated orientation.

**Figure 18:**
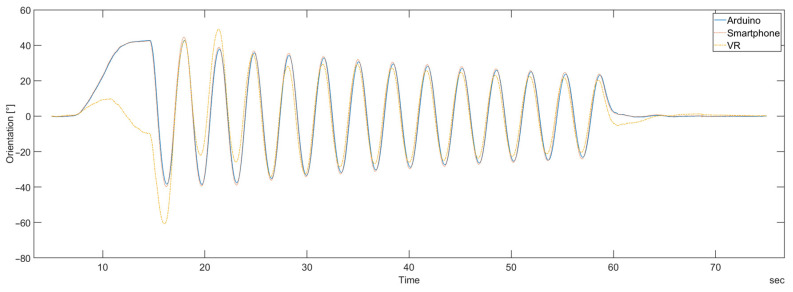
The orientation on the *z*-axis of different types of devices, calculated for the entire duration of a measurement.

Figure 18 shows the results of calculated orientation for the ICMAK Arduino device, the iPhone smartphone, and the VR headset for the entire duration of the measurement. The comparison made for different types of devices revealed an unexpected result regarding the VR device. In the first two periods, its calculated orientation shows a considerable deviation from the orientation obtained for the other two devices.

**Figure 19:**
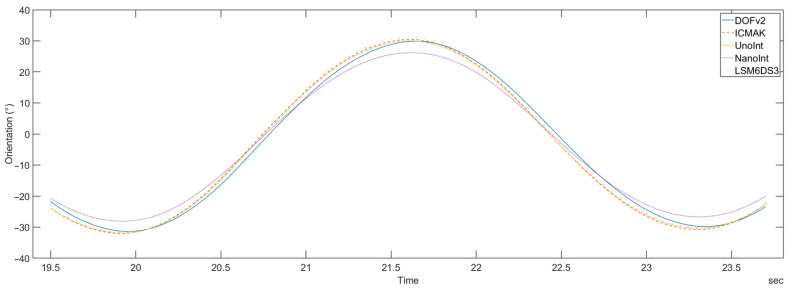
One period of calculated orientation for all five DIY sensors.

For the comparison of sensor performance, a single period of orientation is observed. The calculated orientation for DIY sensors is shown in Figure 19.

It can be observed that the calculated orientation for all five devices is synchronized, and only negligible latency can be detected. The amplitude of orientation for the NanoInt sensor is smaller than the ones observed for the other four sensors, and the UnoInt sensor displays a small time shift, relative to the other four sensors.

**Figure 20:**
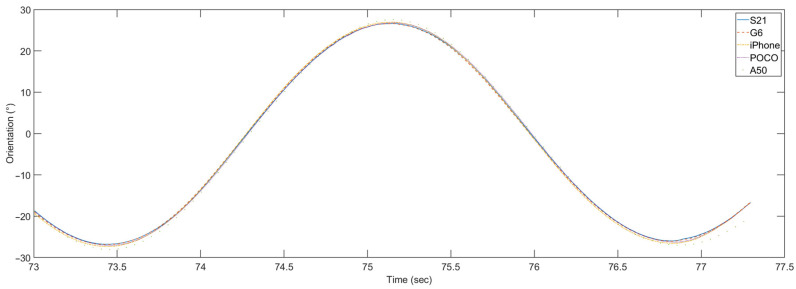
One period of calculated orientation for all five smartphones.

To allow the comparison of the orientation data calculated for smartphones, the gyroscope data obtained from the A50 smartphone had to be time-adjusted in the postprocessing stage. The orientation calculated for all five smartphones after this adjustment is shown in Figure 20.

In this case, the orientation calculated for all five smartphones is in better mutual agreement than it was for the DIY sensors. Only the A50 smartphone shows a deviation in amplitude, compared to all other devices.

**Figure 21:**
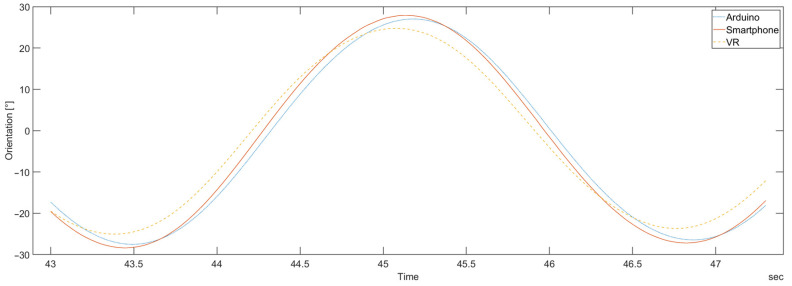
One period of calculated orientation for an Arduino-based device (the ICMAK sensor), a smartphone (the iPhone), and the VR device.

As indicated earlier, the measurements were made in three stages: the DIY sensors were investigated first, then the smartphones, and the comparison of different devices was made at the end. To facilitate the comparison of sensors from different devices, a typical device representative to each group was chosen. The criteria used in the selection process were the smallest signal-to-noise ratio in all axes, the smallest deviation from the rest of the group, and the smallest number of observed issues in the raw data of both sensors (the accelerometer and the gyroscope). Considering all of these criteria together leads to the choice of the most reliable, accurate, and stable devices in their respective groups. In particular, the ICMAK sensor was chosen as the representative of the DIY sensors, and the iPhone was chosen to represent the smartphone group. As the ICMAK sensor is connected to an Arduino microprocessor, it is labeled Arduino, and iPhone is labeled Smartphone. A period of calculated orientation for a comparison of three different types of devices is shown in Figure 21.

It can be observed that the orientation calculated for the DIY and the smartphone device agree quite well, with just a small time offset between the two. Compared to them, the VR device exhibits not only a smaller amplitude of the calculated orientation, but also a time shift.

### 5.2. Measurement Statistics

A basic statistical analysis was performed for four parameters that were measured in this research. As there is no “ground truth” orientation to which the measured data can be compared, the mean value is calculated for every time instant from the measured values obtained from all devices. The calculation was made for all five DIY sensors, as shown in Table 2; for all five smartphones, as shown in Table 3; and as a comparison of a DIY device, a smartphone, and the VR unit, as shown in Table 4. The representative devices from the DIY group and the smartphone group are the same ones as above.

Before calculating the mean value of orientation, the timestamps of the measured data must correspond to each other, and the sampling rate must be made equal for different devices and sensors; that is, the data need to be upsampled and synchronized. Using the calculated mean value as the reference, the standard deviation of the calculated orientation is found for each device or sensor for a single pendulum period (σ_A1_) and for ten consecutive pendulum periods (σ_A10_).

**Figure 22:**
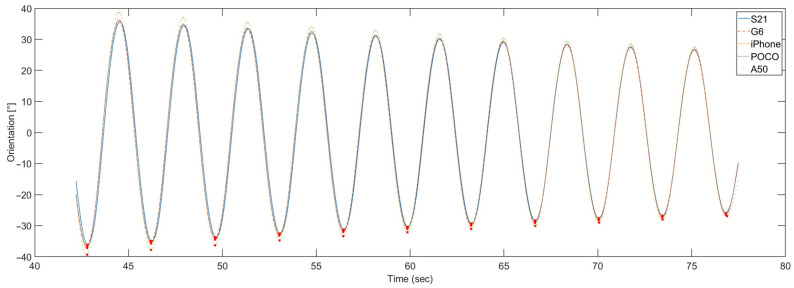
Ten pendulum periods of calculated orientation for all five smartphones.

As neither the length of the pendulum rod nor the total mass changes during the measurements, the actual swing period of the pendulum is constant. Therefore, it is used as the duration reference for the orientation period. The mean value was calculated from ten consecutive periods in a measurement. Figure 22 shows ten consecutive periods of calculated orientation for five smartphones. The orientation minimums, which correspond to one of the maximum deflection points of the pendulum, are marked with red stars.

For each device or sensor, the mean value ($\bar{x_{P}}$) and the standard deviation (σ_P10_) of the duration of the pendulum period were calculated. Built-in MATLAB functions [25] were used for data synchronization and statistical analysis.

The measurement statistics are shown in tables as follows. Table 2 displays the statistics for the orientation data calculated from raw sensor data obtained from DIY devices.

Table 3 displays the statistics for the orientation data calculated from raw data obtained from sensors in smartphones.

Table 4 displays the statistics for the calculated orientation data for the representative devices of all three types.

### 5.3. Discussion

The measurements performed in this research reveal several issues that need to be noted.

The low sample rate of magnetometer sensors (under 20 Hz in some cases) represents a bottleneck for data acquisition for the proposed method. Therefore, the magnetometer data were excluded from the measurements and the orientation calculation. The VR device operates at the lowest sampling frequency of all investigated devices, and it is fixed at 60 Hz. The period of the pendulum used in the proposed measurement setup is 3.4 s. With the maximum deflection angle of ±45 degrees and the maximum measured angular velocity of 50°/s, the fixed sample rate of 60 Hz leads to the measurement uncertainty of 0.78°.

Moreover, the measurement uncertainty depends on the absolute precision of the timestamp that accompanies each measured sensor value. Since each device has its own internal clock of limited accuracy, it is impossible to conclude which device clock is the most accurate one compared to absolute time. Therefore, the timestamps were aligned by using strong mechanical pulses at the start and the end of the measurement, as explained in Section 4.1. With this procedure, a time drift that could occur due to the inaccurate internal clock of a device has been eliminated.

The DIY devices have hardware limitations regarding the communication protocols they use. Some of the investigated devices use only the I2C protocol, some use only the SPI protocol, and some can alternate between them. The authors have decided to use the I2C protocol for all the sensors, where possible (only the UnoInt uses the SPI protocol in this setup). No difference between these protocols was observed regarding the communication speed. Stock libraries were used for all sensors. However, for some sensors the sampling rate enabled by stock libraries was, in fact, lower than the sampling rate provided by alternative, community-written libraries. In some cases, the use of community-written libraries quadrupled the sampling rate and enhanced the time resolution of data acquisition. The NanoInt sensor sometimes exhibits a problem of changing the flag of the status register, even though the data were refreshed. One of the sensors does not have axes labels on its PCB or any markings on the chip itself, making its true orientation ambiguous. This sensor was orientated based on common sense, and the results confirmed that its orientation was properly chosen. The conducted measurements reveal that some DIY sensors have lower resolution (ICMAK), while the highest resolution is exhibited by the DOFv2 sensor.

When comparing smartphones, there was no possibility to manipulate their built-in software. A MATLAB Mobile application [43] was used, and the only parameters the user can control are the sampling frequency and the power state of the sensors used for data acquisition. Although the sampling frequency was set to 100 Hz for all smartphones, the actual sampling rate was inconsistent and generally higher than 100 Hz. The calculated orientation is satisfactory for all smartphones. The lowest accuracy and the highest sampling inconsistency was displayed by the A50 smartphone. This finding is expected, as the A50 is the only lower-budget device within the selected group of smartphones.

The comparison of the representative devices of three different types reveals an inconsistency of the calculated orientation for the VR device, compared to the smartphone and the DIY device. From Figure 18, it is obvious that its orientation curve that was calculated from the read raw IMU data behaves differently. The datasheet of the Unreal engine [26] used for programming a VR device for data acquisition clearly states that the obtained data should be the raw data from sensor outputs. The measurements conducted in this paper disprove this statement if compared to the results obtained from the other two devices. The lack of documentation for the VR device and the fact that the VR device is a closed system prevent further investigation of this behavior.

The results presented in Table 2, Table 3 and Table 4 indicate that the mean period of the pendulum is about 3.4 s. However, the period is the shortest for DIY devices, and it is the longest for smartphones. The period of the pendulum exhibits slight changes due to the shift of its center of mass, as it is not an ideal mathematical pendulum, but a physical one. Although an additional weight of considerable mass is mounted on the bottom of the pendulum, the center of mass of the entire pendulum depends on the mass of the investigated devices mounted on the wooden platform. This does not represent a problem when devices and sensors measured within the same measurement are compared, because the total mass of the setup is constant during the measurement. However, the data obtained from measurements made on different physical setups (different groups of devices with different total mass) are not entirely comparable. The shortest mean period is obtained for DIY sensors due to their small mass (no batteries). The longest mean period is obtained for measurements performed only on battery-powered devices (increased mass due to batteries).

For the DIY devices, the standard deviation for one orientation period (σ_A1_) is higher than the standard deviation for ten periods (σ_A10_). The results obtained for smartphones and for the comparison of all three types of devices show the exact opposite. At first glance, this finding might indicate that the DIY sensors provide the most accurate data out of the three investigated platforms and that the orientation values stabilize as the measurement progresses. However, the proper conclusion is that the DIY devices are the most consistent among themselves out of the three investigated device types. The other two comparisons are influenced by the fact that one of the devices provides orientation data that are inconsistent with the data obtained from other devices. This automatically changes the mean values of orientation taken as reference and increases the dispersion of data expressed with the values of standard deviation.

The results of similar measurements conducted in [36] exhibit the same issues as identified in this paper. The hardware setup also uses Arduino boards as a microcontroller. Measurements performed on DIY sensors suffer from inconsistent sampling frequency from one sensor to the next, which can occur due to the inaccuracies in the microcontroller or a delay in the way the timing is programmed. Raw data obtained from the gyroscope and accelerometer before filtering exhibit the same amplitude irregularities (spikes). The maximum raw data error exceeds 6.5°, which is similar to the error obtained in the presented research, as expressed with the standard deviations listed in Table 2, Table 3 and Table 4.

Although the presented method cannot definitively prove this claim, it is reasonable to assume that the devices and sensors investigated in this research are not applicable for tracking very fast head movement. As shown in [44,45], the maximum expected rotational speed of the human head can exceed 800°/s. The sampling frequencies available in the investigated devices would not facilitate a fine enough angular resolution of the obtained orientation to adequately quantify such extreme motion. The only way to track such fast movement is to use sensors with a higher sampling rate. If the expected head rotation speed is kept below 90°/s [45], the investigated devices and sensors provide the IMU data with a time/angular resolution that is adequate for binaural synthesis without affecting the user experience.

### 5.4. The Strengths and Limitations of the Proposed Method

The proposed measurement method and the corresponding measurement setup allow the user to investigate different types of devices and sensors simultaneously, thus enabling a direct comparison of the measured data. Various devices can be mounted onto the test platform in arbitrary orientations. The process of time synchronization between the devices is simple and accurate, as shown above. Although the system enables dynamic measurements with a great range of motion, the devices themselves are firmly fixed to the pendulum as part of the measurement setup. Therefore, wired communication and the power supply can be used, as the pivot point of the pendulum is static, and all the wiring can lead through this point and not be damaged. Wired communication facilitates robust data transfer with minimal latency. As there are no parts of the measurement setup that generate magnetic disturbance, magnetometer sensors can be investigated as well. The setup itself is cost-saving and easy to assemble.

The length of the pendulum rod determines the magnitude of acceleration and the period of oscillation. A longer rod increases the measurement range, leading to a higher quality of measurements. However, the length of the rod is limited by the ceiling height and the amount of available space in the plane the pendulum swings in. Moreover, a longer rod length leads to stronger torsional forces that act on the wooden platform the sensors are mounted on. As a result, strong wobbling is present, and it was recorded for all measured devices, as shown in Figure 15.

For most devices, there is no official documentation or data regarding the position and the orientation of the IMU sensors built into them. This represents an issue regarding the determination of the exact measurement position and orientation for each device on the test platform. Incorrect positioning of a device on the test platform leads to the misalignment of the equilibrium position of the pendulum with the one of the sensor itself, and the actual rotation radius from the pivot point to the sensor is compromised. This problem leads to corrupt data and represents an issue that remains to be solved.

The proposed measurement setup relies on the free, gravity-induced motion of a pendulum along a circular arc. The raw gyroscope data show that the angular velocity that can be achieved using this setup is not high enough to allow the assessment of the investigated devices as possible head trackers for applications that include extreme head movement. The setup can be used for such an assessment where applications that require moderate or mild head movement are concerned.

## 6. Conclusions

This research focused on measuring the performance of IMU sensors on various hardware platforms that are widely used for binaural head-tracking applications, either as DIY projects or as part of commercial headsets. IMU sensors must provide accurate data for the calculation of the orientation of the listener’s head over time, so that the correct HRTFs can be chosen in the binaural synthesis process. While preparing the optimal measurement setup that can be adapted to all measured hardware platforms, many challenges described in this paper had to be solved to obtain accurate and directly comparable results. This includes the acquisition of IMU data from the same sensor type from all devices, the unification of their sampling frequencies, the synchronization of their internal time bases, and the programming of a common algorithm for the calculation of the orientation as the ultimate comparison parameter for all platforms.

All measured devices and sensors provided raw data with minimal latency. For DIY devices based on the Arduino platform, the highest latency is displayed by the DOFv2 sensor, which also exhibits the highest quantization resolution among all DIY sensors. The DOFv2 sensor proved to be the most suitable for applications that require the detection of small changes in orientation. The ICMAK sensor is the most suitable for applications where fast orientation changes must be detected. The sampling rate of the DIY sensors is essentially constant, with only minor oscillations. It can be adjusted and increased by using non-official libraries available from various community-based repositories.

The measurements performed on smartphones proved that these devices can be used for collecting the data provided by their built-in gyroscopes and accelerometers. Several products are already available that enable a smartphone to be mounted as a headset. In this case, the internal IMU sensors in smartphones are used to facilitate the creation of an immersive virtual reality. The measurements have shown that most smartphones have a satisfactory data output without noticeable latency, as the key prerequisite for creating virtual reality environments. The cheapest smartphone in the testing group proved to be the least accurate one.

The comparison of devices of different types included one DIY device, one smartphone, and the VR headset. The results have shown that there is a strong agreement between the orientation data calculated for the DIY device and the smartphone device, whereas the orientation data of the VR headset show a mismatch with the other two device types. Additionally, the data from the VR headset are preprocessed before being sent to the user. This means that the VR device, being a closed system, works well in its own applications. On the other hand, its use as the source of raw data for external calculations of its orientation for other uses is questionable at best. This is especially true if we consider that some VR headsets also use cameras mounted on the headset to increase the accuracy of the orientation calculated internally using sensor fusion algorithms, but this goes beyond the scope of this research.

In further research using this method, an additional high-quality sensor will be mounted on the pendulum. As a device with a high sampling rate and accuracy, it will serve as ground truth, i.e., the reference for measurements. The research team is also considering adding a sensor that would track the angle inclination of the pendulum and measure the change of angular velocity over the period of the pendulum.

## Figures and Tables

**Table 1:** Overview of previous measurements on IMU sensors as pilot studies for the research presented in this paper.

Previous Research	Main Objectives
Research framework [20]	Research hypothesis, testing of IMU sensor connectivity and data-transfer speed.
Static measurements [31]	IMU sensor data drift detection and evaluation in static state, quality comparison between different devices.
Dynamic measurements [30]	Pilot measurements of various IMU devices on rotary platforms with variable rotation speed, tuning of dynamic measurement parameters.

**Table 2:** Statistical analysis of the orientation data calculated from raw sensor data obtained from DIY devices.

Parameter	DOFv2	ICMAK	UnoInt	NanoInt	LSM6DS3
σ_A1_ (°)	6.2575	6.3687	6.3156	5.5542	6.3031
σ_A10_ (°)	5.4147	5.4754	5.4414	4.7898	5.5059
$\bar{x_{P}}$ (s)	3.361	3.361	3.361	3.361	3.362
σ_P10_ (s)	0.0066	0.0117	0.0088	0.0108	0.0082

**Table 3:** Statistical analysis of the orientation data calculated from raw sensor data obtained from smartphones.

Parameter	S21	G6	iPhone	POCO	A50
σ_A1_ (°)	5.3535	5.3696	5.4540	5.3988	5.7005
σ_A10_ (°)	7.3559	7.3386	7.4302	7.3641	8.0204
$\bar{x_{P}}$ (s)	3.401	3.403	3.404	3.404	3.409
σ_P10_ (s)	0.0160	0.0144	0.0133	0.0126	0.0149

**Table 4:** Statistics for the orientation data calculated for the representative devices of all three types.

Parameter	Arduino	Smartphone	VR
σ_A1_ (°)	5.5712	5.6395	5.1424
σ_A10_ (°)	7.6047	7.6697	7.0061
$\bar{x_{P}}$ (s)	3.387	3.384	3.381
σ_P10_ (s)	0.0204	0.0153	0.0243

## Data Availability

All data can be obtained from the authors upon request.
